# The Effect of tRNA^[Ser]Sec^ Isopentenylation on Selenoprotein Expression

**DOI:** 10.3390/ijms222111454

**Published:** 2021-10-23

**Authors:** Noelia Fradejas-Villar, Simon Bohleber, Wenchao Zhao, Uschi Reuter, Annika Kotter, Mark Helm, Rainer Knoll, Robert McFarland, Robert W. Taylor, Yufeng Mo, Kenjyo Miyauchi, Yuriko Sakaguchi, Tsutomu Suzuki, Ulrich Schweizer

**Affiliations:** 1 Institut für Biochemie und Molekularbiologie, Rheinische Friedrich-Wilhelms-Universität Bonn, D-53115 Bonn, Germany; fradejan@uni-bonn.de (N.F.-V.); sbohl@uni-bonn.de (S.B.); wenchao.zhao@ki.se (W.Z.); ureuter@uni-bonn.de (U.R.); rainer.knoll@uni-bonn.de (R.K.); 2 Institute of Pharmacy and Biochemistry, Johannes Gutenberg University of Mainz, Staudingerweg 5, D-55128 Mainz, Germany; akotter@uni-mainz.de (A.K.); mhelm@uni-mainz.de (M.H.); 3 Wellcome Centre for Mitochondrial Research, Clinical and Translational Research Institute, Faculty of Medical Sciences, Newcastle University, Newcastle upon Tyne NE2 4HH, UK; robert.mcfarland@ncl.ac.uk (R.M.); robert.taylor@ncl.ac.uk (R.W.T.); 4 Department of Chemistry and Biotechnology, Graduate School of Engineering, University of Tokyo, Tokyo 113-8656, Japan; moyufeng@g.ecc.u-tokyo.ac.jp (Y.M.); mkenjyo@chembio.t.u-tokyo.ac.jp (K.M.); yurikos@chembio.t.u-tokyo.ac.jp (Y.S.); ts@chembio.t.u-tokyo.ac.jp (T.S.)

**Keywords:** *Trit1*, isopentenylation, tRNA^[Ser]Sec^, selenoproteins

## Abstract

Transfer RNA^[Ser]Sec^ carries multiple post-transcriptional modifications. The A37G mutation in tRNA^[Ser]Sec^ abrogates isopentenylation of base 37 and has a profound effect on selenoprotein expression in mice. Patients with a homozygous pathogenic p.R323Q variant in tRNA-isopentenyl-transferase (*TRIT1*) show a severe neurological disorder, and hence we wondered whether selenoprotein expression was impaired. Patient fibroblasts with the homozygous p.R323Q variant did not show a general decrease in selenoprotein expression. However, recombinant human TRIT1^R323Q^ had significantly diminished activities towards several tRNA substrates in vitro. We thus engineered mice conditionally deficient in *Trit1* in hepatocytes and neurons. Mass-spectrometry revealed that hypermodification of U_34_ to mcm^5^Um occurs independently of isopentenylation of A_37_ in tRNA^[Ser]Sec^. Western blotting and ^75^Se metabolic labeling showed only moderate effects on selenoprotein levels and ^75^Se incorporation. A detailed analysis of *Trit1*-deficient liver using ribosomal profiling demonstrated that UGA/Sec re-coding was moderately affected in *Selenop*, *Txnrd1*, and *Sephs2*, but not in *Gpx1*. 2′O-methylation of U_34_ in tRNA^[Ser]Sec^ depends on FTSJ1, but does not affect UGA/Sec re-coding in selenoprotein translation. Taken together, our results show that a lack of isopentenylation of tRNA^[Ser]Sec^ affects UGA/Sec read-through but differs from a A37G mutation.

## 1. Introduction

Selenoproteins are proteins containing the rare and essential amino acid selenocysteine (Sec), which is co-translationally inserted into proteins. Hierarchical expression of selenoproteins depends on the availability of selenium (Se) both among organs and among individual selenoproteins [1]. Moreover, at lower Se availability, selenoprotein expression is more robust in female than in male mammals [2]. The hierarchy among organs is established by provision of selenoprotein P (SELENOP) by the liver and its receptor-mediated uptake through endocytic receptors [3,4]. Several mechanisms cooperate to establish a second hierarchy among selenoproteins in one cell. For example, glutathione peroxidase 1 (GPX1) and SELENOW levels closely reflect bioavailability of Se, while GPX4 and thioredoxin reductases (TXNRD) remain stably expressed at lower Se levels. This hierarchy has been correlated with the affinities of selenocysteine insertion sequences (SECIS) present in the 3′-untranslated regions of selenoprotein mRNAs to the SECIS-binding protein 2 (SECISBP2) [5,6,7]. These correlations, however, are not perfect, and binding and competition of other mRNA binding proteins such as RPL30, NUCLEOLIN, and eIF4A3 have been invoked to explain aspects of the hierarchy [8,9,10]. Moreover, selenoprotein mRNAs may be subject to mRNA surveillance pathways if Se levels are limiting, in particular, *GPX1* and *SELENOW*.

**Figure 1:**
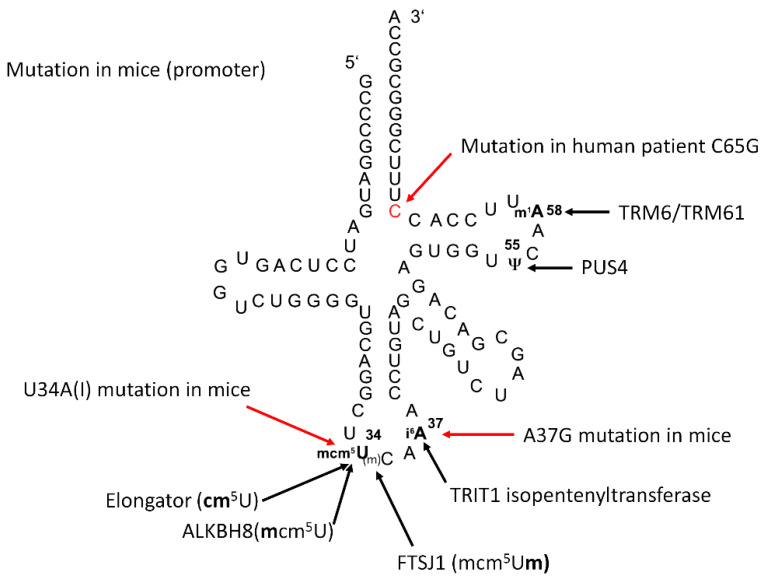
Modifications and mutations in tRNA^[Ser]Sec^. Sequence of murine tRNA^[Ser]Sec^ with post-transcriptional modifications indicated [20]. Where proposed, we mentioned the respective enzymes responsible for the modifications (black). Labelled in red are mutations in the primary sequence of tRNA^[Ser]Sec^ in transgenic mouse models or observed in a human patient. The U34A mutant tRNA^[Ser]Sec^ is further deaminated in vivo to inosine (I).

Sec is encoded by the UGA codon, and thus translation involves a competition between elongation and termination. Central to this process is tRNA^[Ser]Sec^. This tRNA was discovered as a rare seryl-tRNA that recognizes a UGA codon [11,12]. Accordingly, it is amino-acylated by SerRS. The 3′-Ser is subsequently phosphorylated by PSTK and further converted to Sec-tRNA^[Ser]Sec^ by selenocysteine synthase [13,14,15,16]. Unlike other tRNAs, there is only one gene encoding tRNA^[Ser]Sec^ in mammals, *Trsp* (in mice), and *TRU-TCA1-1* (in humans). Transfer RNA^[Ser]Sec^ carries several modifications (Figure 1). In both bacteria and in vertebrates, the anticodon loop carries a hypermodified 5-methylcarboxymethyl (mcm^5^)U_34_, which may be further methylated on the 2′O-position of the ribose (mcm^5^Um_34_), and a *N^6^*-isopentenyl(i^6^)A_37_ [17,18,19,20,21].

Mutations in tRNA^[Ser]Sec^ affect selenoprotein expression [22]. In transgenic mice, expression of a hypomorphic tRNA^[Ser]Sec^ with a promotor mutation leads to a neurological phenotype [23], and gene targeting of *Trsp* leads to complete abrogation of selenoprotein expression [24,25,26]. A pathogenic homozygous c.C65G variant in *TRU-TCA1-1* causes a phenotype resembling the phenotype of patients with pathogenic *SECISBP2* variants [27,28,29]. Thus, the level and integrity of tRNA^[Ser]Sec^ modulates selenoprotein expression. Interestingly, mutations in A_37_ and U_34_ in tRNA^[Ser]Sec^ affect not only the levels but also the hierarchy of selenoprotein expression. Both mutations reduce expression of GPX1 effectively, while GPX4 and TNXRD1 remain more stably expressed [30,31]. Another publication presented data that support the notion that the A37G mutation acts, in part, as a dominant negative [32]. In a pioneering study interrogating selenoprotein translation using ribosomal profiling, it was shown that Se availability modulates the efficiency of UGA/Sec recoding [33]. This study further showed that the A37G mutant tRNA^[Ser]Sec^ was not efficiently supporting selenoprotein translation, even in the presence of supra-nutritional selenium. Therefore, it is evident that modification of tRNA^[Ser]Sec^ has a major impact on the process of UGA/Sec re-coding. In fact, the crystal structure of hypomodified tRNA^[Ser]Sec^ showed a disordered anticodon stem loop [34]. In the cryo-EM structure of a bacterial ribosome in complex with mRNA and elongation factor SELB, the modified tRNA^[Ser]Sec^ shows stacking of i^6^A_37_ on the anticodon:codon minihelix; however, modification of U_34_ was not resolved [35,36].

It has been observed that 2′O-methylation of mcm^5^U_34_ (mcm^5^Um) in tRNA^[Ser]Sec^ correlates with Se bioavailability [19,37]. Due to the correlation with hierarchical selenoprotein expression, a role for tRNA modification was proposed, and the effect of A37G and U34A(I) mutations were explained with the lack of 2′O-methylation of nucleoside 34 in both mutant tRNAs [30]. Interference with 5-methylcarboxymethylation of tRNA^[Ser]Sec^ by mutation of the enzyme ALKBH8 reduced selenoprotein expression, supporting a role for U_34_ modification in UGA/Sec recoding [38,39].

The observation that treatment with lovastatin affected selenoprotein expression in cultured cells suggested that isopentenylation of tRNA^[Ser]Sec^ was important for its function [40,41]. Later, it was shown that tRNA-isopentenyltransferase (TRIT1) was the enzyme modifying tRNA^[Ser]Sec^, and knock-down of *Trit1* reduced GPX1 expression in NIH 3T3 cells under the condition of low Se availability [42]. Patients carrying pathogenic variants in *TRIT1* show microcephaly with epilepsy that was primarily explained by a mitochondrial disease associated with deficient isopentenylation of mitochondrial tRNAs [21,43]. Since neurological disorders including seizures are also phenotypes observed in several mouse models carrying mutations in tRNA^[Ser]Sec^ [23,44], we wondered whether selenoprotein expression was also affected in patients harboring pathogenic *TRIT1* variants.

We therefore studied selenoprotein biosynthesis in *TRIT1*-deficient human fibroblasts, recombinant human TRIT1, and in mice with inactivation of *Trit1*.

## 2. Results

### 2.1. TRIT1-Mutant Human Fibroblasts

**Figure 2:**
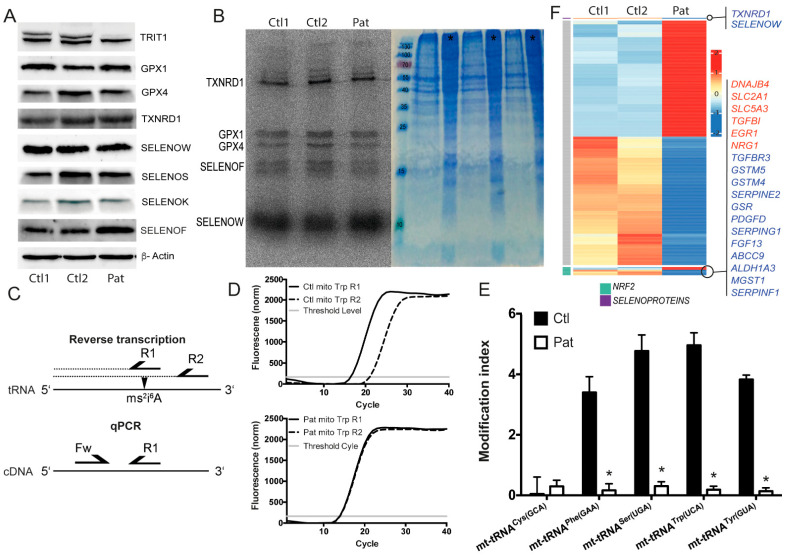
Selenoprotein expression in patient fibroblasts carrying a pathogenic homozygous TRIT1^R323Q^ variant. (**A**) Western blot comparing selenoprotein expression in *TRIT1* patient fibroblasts with two control fibroblast lines. The signal corresponding to TRIT1 protein is reduced almost to the detection limit in the *TRIT1* patient cells, while the unspecific (lower) band suggests equal loading. β-Actin served as control. (**B**) Metabolic ^75^Se-labeling of cultured fibroblasts reveals normal ^75^Se incorporation in selenoproteins. Coomassie brilliant blue stained gel shows equal protein loading. Asterisks represent wells loaded with un-labelled protein to avoid diffusion (**C**) RT-PCR to determined ms^2^i^6^A in tRNAs. The two steps, reverse transcription of tRNA and qPCR of cDNA, are depicted. Primers are represented as half arrows (R1 and R2 are reverse primers and Fw is the forward primer) Arrowhead shows the position of the ms^2^i^6^A. (**D**) Determination of tRNA modification index based on RT-PCR. Traces from mt-tRNA^Trp^ analysis. (**E**) Modification index of several mt-tRNAs normally containing ms^2^i^6^A_37_ modifications depends on functional TRIT1. (**F**) Heatmap of significantly regulated genes from human fibroblasts focused on selenoprotein and NRF2 target genes. Up-regulated and down-regulated genes in the patient fibroblasts are depicted in red and blue, respectively.

Studies of Kim and colleagues suggested that the acquisition of post-transcriptional modifications in tRNA^[Ser]Sec^ was sequential and interdependent in *Xenopus* oocytes [20]. Likewise, profound changes in selenoprotein expression were described in mouse models, wherein A_37_ in tRNA^[Ser]Sec^ was not isopentenylated due to a A37G mutation [30]. Hence, we wondered whether fibroblasts derived from a patient carrying a homozygous pathogenic variant in *TRIT1* represented an excellent model to study the role of i^6^A in tRNA^[Ser]Sec^ for selenoprotein expression [43]. To our surprise, Western blot against selenoproteins did not reveal any reduction in the patient fibroblasts (Figure 2A), despite the fact that the TRIT1 protein appeared greatly reduced. We subsequently metabolically labelled the fibroblasts with ^75^Se-selenite, finding no reduction in ^75^Se incorporation into selenoproteins (Figure 2B). We then asked whether another unidentified A_37_-tRNA-isopentenyltransferase activity was expressed in these cells. We therefore determined the modification indices of several tRNAs that are normally isopentenylated, using an established RT-qPCR technique [45]. This assay exploits the sensitivity of a reverse transcriptase reaction to the presence of 2-methylthio-i^6^A (ms^2^i^6^A) in the tRNA substrate. The resulting cDNA is then quantified by qPCR (Figure 2C). This technique independently confirmed the results obtained before with a positive hybridization assay [43] and showed that those tRNAs that are normally containing ms^2^i^6^A_37_ are hypomodified in *TRIT1*-mutant cells (Figure 2D,E). In order to obtain an overview of the gene regulation of selenoproteins and NRF2-depedent anti-oxidative genes, we performed RNA sequencing in *TRIT1*-mutant fibroblasts (Figure 2F). Some NRF2 target genes were up-regulated (e.g., *MT2*), but others were down-regulated (e.g., *GSTM4, GSTM5, MGST1*). Induction of mitochondrial transcripts is in line with the mitochondriopathy of the patient. Among selenoproteins, only *TXNRD1* and *SELENOW* were decreased at the mRNA level, but this was not reflected at the protein level (Figure 2A), suggesting that reduced mRNAs are a result of gene-specific regulation rather than an effect on selenoprotein translation. Taken together, the p.R322Q variant in *TRIT1* did not show a general deficiency in the function of tRNA^[Ser]Sec^ in selenoprotein translation.

### 2.2. In Vitro Activity of TRIT1 and TRIT1R323Q

**Figure 3:**
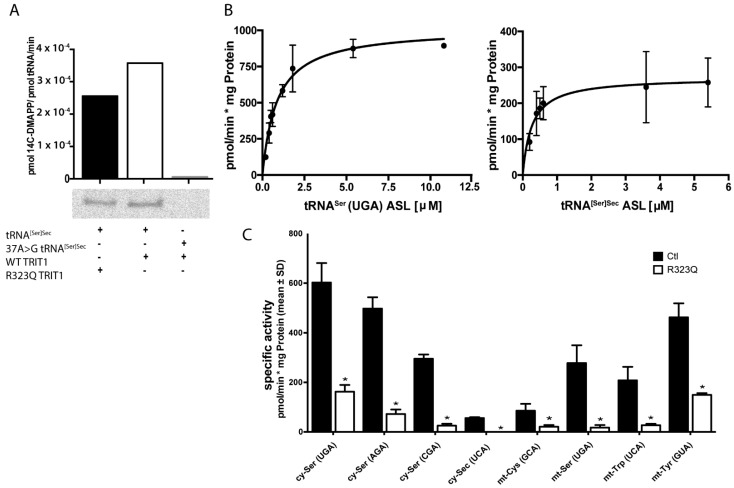
Activity assays using recombinant TRIT1. (**A**) In vitro assay using wild type and p.R323Q variant TRIT1 recombinant proteins and in vitro transcribed tRNA^[Ser]Sec^. Isopentenylated tRNA was also detected in a urea-acrylamide gel. (**B**) Representative results of kinetic analyses of TRIT1 with ACF substrates corresponding to mt-tRNA^Ser^_(UGA)_ and tRNA^[Ser]Sec^. (**C**) Specific activities determined for eight substrates using TRIT1 (Ctl) and p.R323Q. N = 3. * *p* < 0.05, Student’s *t*-test. The ACF oligonucleotide concentration in the endpoint assay corresponded to the K_M_ of the oligonucleotide with the wild-type enzyme (Table 1).

The exact function of Arg323 in human TRIT1 is not known, but a crystal structure of the yeast tRNA-isopentenyltransferase MOD5 suggested that the amino acid is involved in substrate binding [36,46]. Thus, we wondered whether Arg323 might interact only with some, but not all substrates, and a substitution to Gln might specifically not affect isopentenylation of tRNA^[Ser]Sec^. We therefore recombinantly expressed human TRIT1 protein along with a p.R323Q variant and subjected the purified proteins to biochemical activity assays. Recombinant TRIT1 with and without the p.R323Q variant transferred ^14^C-labelled dimethylallylpyrophosphate (DMAPP) to in vitro transcribed (IVT) tRNA^[Ser]Sec^, while a tRNA^[Ser]Sec^ mutant with A37 replaced by G was not isopentenylated, as expected (Figure 3A). In order to test a battery of cytosolic and mitochondrial tRNAs in the following isopentenylation assays, we used synthetic anticodon-containing fragment (ACF) oligonucleotides as substrates in a filter-binding assay. As a first step, we determined for each substrate the K_M_ values along with the respective V_max_ towards recombinant human TRIT1 (Figure 3B; Table 1). In order to assess the effect of the p.R323Q variant, we then determined in a separate experiment the specific activities of recombinant TRIT1 and the p.R323Q variant protein against eight ACF substrates (Figure 3C). The variant protein was significantly less active towards each four mitochondrial and four cytosolic tRNA ACF substrates, including tRNA^[Ser]Sec^. This finding suggests that the p.R323Q variant affects activity towards all tRNA substrates.

### 2.3. Inactivation of Trit1 in the Mouse

**Figure 4:**
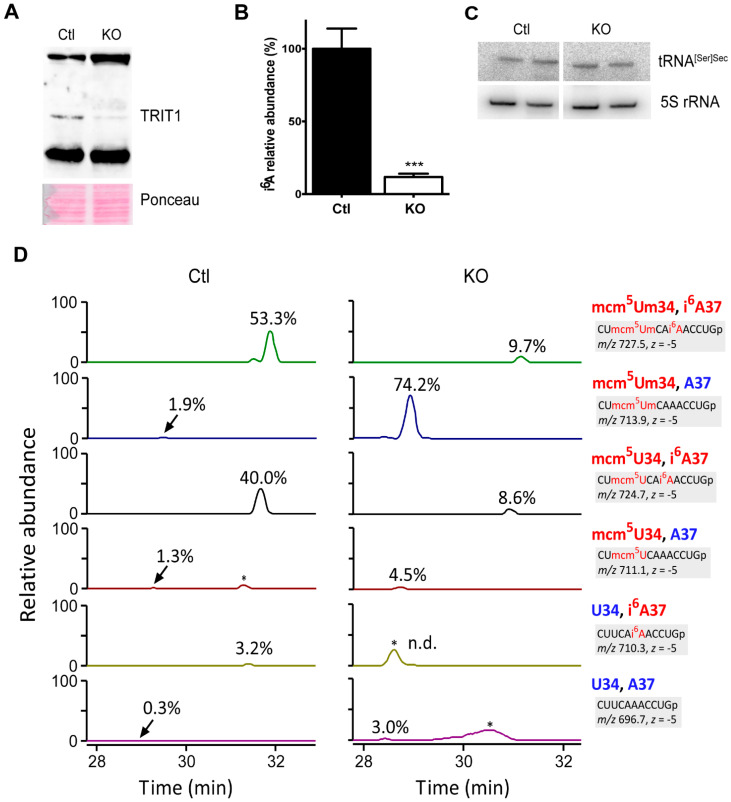
Knockout of *Trit1* in liver abrogates formation of i^6^A in tRNA and tRNA^[Ser]Sec^ isopentenylation. (**A**) Western blot on liver extract using an antibody against TRIT1. (**B**) Levels of i^6^A in the tRNA fraction isolated from liver are significantly reduced in *Trit1* KO. N = 3. *** *p* < 0.001, Student’s *t*-test. (**C**) Northern blot against tRNA^[Ser]Sec^ and 5S rRNA as control. (**D**) Mass spectrometric analysis of the tRNA^[Ser]Sec^ isolated from Ctl (left panels) and *Trit1* KO (right panels) livers. Each panel from top to bottom shows an extracted-ion chromatogram for the RNase T1-digested anticodon-containing fragments with different modification status at positions 34 and 37. Modification status, sequence of the fragment, *m*/*z* value, and charge state (*z*) are shown on the right for each panel. Relative abundance of each fragment is denoted in each panel. Non-specific peaks are marked with asterisks.

The severity of missense mutations in the selenoprotein biosynthesis pathway may depend on the cell type [47]. Hence, we created conditional *Trit1*-knockout mice and crossed them with an *Alb-Cre* transgene abrogating *Trit1* expression in hepatocytes, an established model for selenoprotein expression analyses. Western blot against TRIT1 shows a greatly diminished signal in livers from *Alb-Cre; Trit1^fl/fl^* (KO) mice (Figure 4A). Accordingly, the abundance of i^6^A in the tRNA fraction isolated from *Trit1* KO liver was less than 10% of the controls (Ctl) compatible with preserved TRIT1 expression in endothelial cells and liver macrophages (Figure 4B). Northern blot against tRNA^[Ser]Sec^ demonstrated unchanged levels in the *Trit1* KO (Figure 4C). We then specifically isolated tRNA^[Ser]Sec^ from *Trit1* KO and Ctl livers by reciprocal circulating chromatography [48], followed by RNase T1 digestion, and subjected the fragments to capillary LC/nanoESI mass spectrometry to analyze its tRNA modifications [49,50]. In Ctl liver, we detected several species of the anticodon-containing fragments with different modification status (Figure 4D). The fully modified fragment with mcm^5^Um at position 34 and i^6^A at position 37 is a major fragment (53.3%), and the same fragment with mcm^5^U at position 34 and i^6^A at position 37 is the second major fragment (40.0%). In *Trit1* KO, both fragments decreased significantly, and instead, the hypomodified fragment with mcm^5^Um_34_ and A_37_ increased drastically (74.2%). The result demonstrated that TRIT1 is responsible for i^6^A_37_ formation in tRNA^[Ser]Sec^. In addition, 5-methylcarboxymethylation of U_34_ does not require prior i^6^A modification. Curiously, the hypomodified fragment with mcm^5^U_34_ and A_37_ was not accumulated in *Trit1* KO (Figure 4D), indicating that 2′O-methylation of mcm^5^Um_34_ is promoted in the absence of i^6^A_37_. In other words, i^6^A_37_ might have an inhibitory effect on FTSJ1-mediated 2′O-methylation. Importantly, although previous studies using A37G mutant tRNA^[Ser]Sec^ suggested that Um_34_ formation depended on prior i^6^A_37_ formation [20,30], our data clearly showed that mcm^5^Um_34_ formation was promoted in the absence of i^6^A_37_ (Figure 4D). Thus, it appears as if the mutant G37 nucleotide in the transgenic mouse model prevented Um_34_ formation and not the lack of isopentenylation of A_37_.

### 2.4. Selenoprotein Expression in Trit1-KO Mice

**Figure 5:**
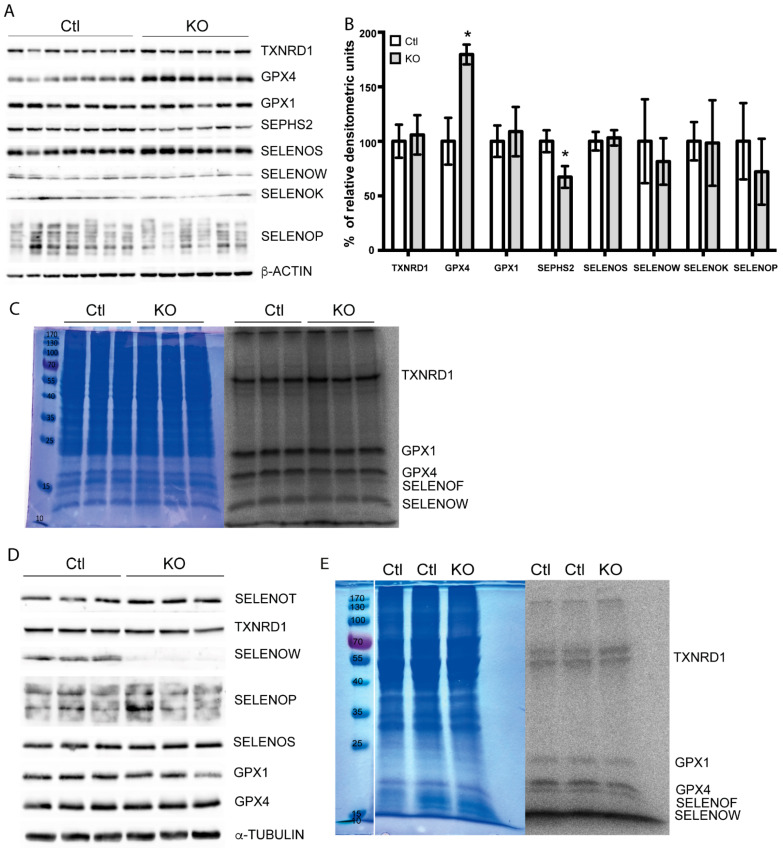
Selenoprotein expression in *Trit1*-knockout (KO) mice. (**A**) Western blot against a panel of 8 selenoproteins in mouse liver. N = 6–7 individual mice. Liver protein, 50 µg, separated on SDS-PAGE. (**B**) Densitometric analysis of the western blot in (**A**). Ponceau was used for normalization. Results are expressed as mean ± SD of the percentage relative to the control (Ctl). GPX4 and SEPHS2 showed significant differences according two-tailed *t*-test. * *p* < 0.05. *p*-values of GPX4 and SEPHS2 were 4 × 10^−6^ and 2.25 × 10^−4^, respectively. (**C**) Metabolic labeling with ^75^Se-selenite of isolated primary hepatocytes. Coomassie brilliant blue-stained gel for loading control (left) and autoradiogram (right). N = 3 individual cultures. (**D**) Selenoprotein western blot from cortices of neuron-specific *Trit1* KO mice. (**E**) ^75^Se-labeling of *Trit1* KO and Ctl neuron cultures (a representative experiment). Coomassie showed equal loading.

Being confident that TRIT1 is the only available tRNA-isopentenyltransferase in mouse hepatocytes and having ascertained that *Trit1* was quantitatively inactivated in our mouse model, we returned to the question whether the hierarchy of selenoprotein expression in hepatocytes depends on tRNA^[Ser]Sec^ A_37_ isopentenylation. To assess the expression of selenoproteins by Western blotting, we focused on those selenoproteins that are easily detected with a panel of antibodies that work well in our hands. There was no general reduction in selenoprotein expression in *Trit1* KO mouse liver, as studied by Western blot against eight selenoproteins (Figure 5A). In particular, GPX1 and SELENOW, which are known to respond sensitively to changes in Se availability, were not changed. In contrast, GPX4 was increased, and SEPHS2 was reduced, as confirmed by densitometric analysis of Western- blots (Figure 5B). Metabolic labeling of primary hepatocytes from wild-type and *Trit1* KO mice did not show diminished ^75^Se incorporation into selenoproteins (Figure 5C). Since selenoprotein expression is organ-dependent, we also tested selenoprotein expression in the brain by Western blot. In neuron-specific *Trit1* KO brains, we detected a reduction in SELENOW, but not of any other selenoproteins (Figure 5D). This suggested that the regulation of SELENOW was gene-specific and not a general effect on selenoproteins. In order to directly assess the neuronal Sec-incorporation machinery, we isolated primary cortical neurons from newborn mice and metabolically labeled them in vitro with ^75^Se. Again, there was no change of ^75^Se incorporation into proteins (Figure 5E). We thus have to conclude that there is no general defect in selenoprotein expression, if tRNA^[Ser]Sec^ lacks i^6^A.

### 2.5. Ribosomal Profiling for Selenoproteins in Trit1-KO

**Figure 6:**
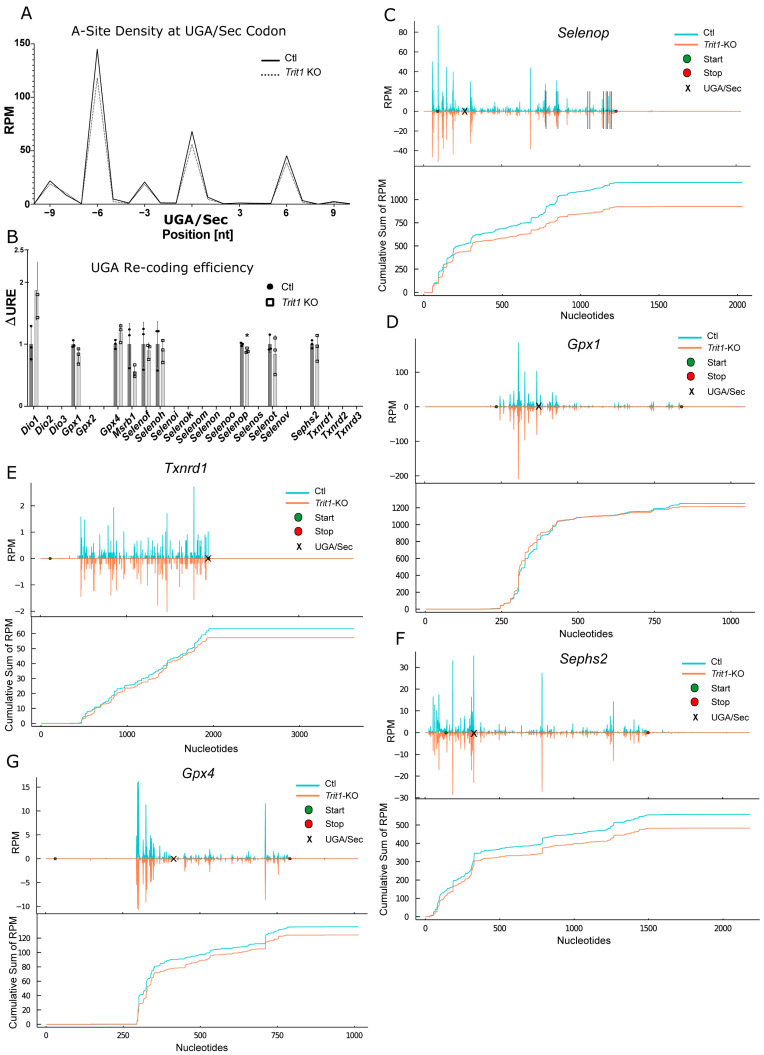
Selenoprotein RiboSeq analysis of *Trit1* knockout (KO) liver. (**A**) RPFs with the UGA/Sec in the A-site expressed as reads per million mapped reads (RPM) over all selenoproteins. (**B**) UGA recoding efficiency (URE, 3′RPF/5′RPF) calculated for selenoproteins with UGA/Sec far from the termination codon. ΔURE is calculated as URE(KO)/URE(Ctl). (**C**–**G**) RPF coverage of selected selenoprotein mRNAs in *Trit1* KO mouse liver. The mean values of the groups were plotted. Start and stop positions are marked as green and red circles. Reads are plotted in blue for control (Ctl) and in orange for *Trit1* KO livers. The position of the UGA/Sec codon is indicated by a black “x” mark. In the case of *Selenop*, following UGA codons after the first are displayed as black vertical lines. Cumulative sums of RPF are shown below the corresponding profiles. RPM: reads per million mapped reads.

We reasoned, that moderate effects on UGA/Sec re-coding may be better revealed by ribosomal profiling in *Trit1* KO mouse liver. Thus, we isolated polysomes from *Trit1* KO and Ctl livers and performed ribosomal profiling. When we plotted all ribosome-protected fragments (RPF) associated with all selenoprotein transcripts around the UGA/Sec codon, we noticed in the *Trit1* KO liver a small reduction in ribosomes sitting with the A-site on the UGA/Sec codon (Figure 6A). Based on a footprint size of 28 nucleotides, these ribosomes mostly represented ribosomes with a tRNA in the A-site. We then calculated the differential UGA re-coding efficiency (ΔURE) for individual selenoproteins, a measure that represents how a condition affects UGA/Sec re-coding in a given selenoprotein [47,51]. According to ΔUGA, effects on selenoprotein translation seemed rather mild; just for *Selenop*, there was a significant change (Figure 6B). SELENOP is unique among mammalian selenoproteins for containing more than one Sec codon per polypeptide. Inspection of the ribosomal coverage along the mRNA revealed a reduced density 3′ of the first UGA/Sec codon (Figure 6C). Similarly, a cumulative sum plot supported this finding in the *Trit1*-KO liver. In contrast to our expectations, no such effect was seen for *Gpx1* whatsoever (Figure 6D). Because the UGA/Sec codon resides in the penultimate position of the *Txnrd1* mRNA, ΔURE cannot be calculated for this selenoprotein. In the ribosomal coverage and cumulative sum plots, however, an impairment of UGA/Sec recoding was apparent (Figure 6E). In Figure 5A,B, SEPHS2 was clearly reduced in *Trit1*-KO liver. Similarly, UGA/Sec re-coding in *Sephs2* was reduced according to the ribosomal coverage and cumulative sum plots (Figure 6F). In agreement with higher protein amounts, we observed a slightly higher coverage on *Gpx4* after the UGA/Sec in the *Trit1* KO compared to Ctl (Figure 6G). Thus, under conditions of adequate dietary Se supply, only moderate effects were found on selenoprotein expression, when tRNA^[Ser]Sec^ was lacking the i^6^A_37_ modification.

### 2.6. Effect of 2′O-Methylation of U_34_ in tRNA^[Ser]Sec^

**Figure 7:**
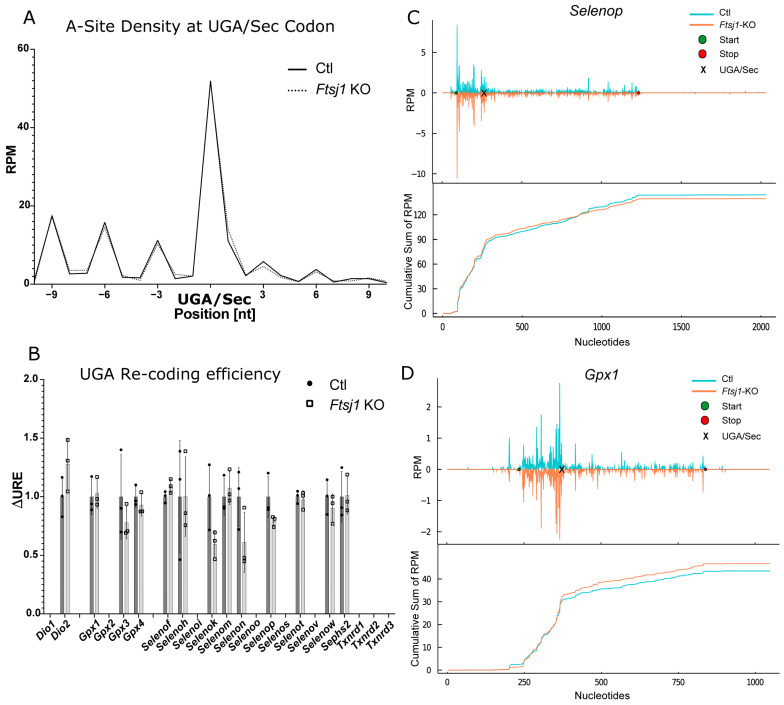
Re-analysis of *Fstj1* knockout (KO) brain RiboSeq data focussed on selenoproteins. (**A**) RPFs with the UGA/Sec in the A-site expressed as reads per million mapped reads (RPM) over all selenoproteins. (**B**) UGA recoding efficiency (URE, 3′RPF/5′RPF) calculated for selenoproteins with UGA/Sec far from the termination codon. ΔURE is calculated as URE(KO)/URE(Ctl). (**C**,**D**) RPF coverage of selected selenoprotein mRNAs in *Ftsj1* KO mouse brain. The mean values of the groups were plotted. Start and stop positions are marked as green and red circles. Reads are plotted in blue for control (Ctl) and in orange for *Ftsj1* KO brains. The position of the UGA/Sec codon is indicated by a black “x” mark. Cumulative sums of RPF are shown below the corresponding profiles. RPM: reads per million mapped reads.

It has been proposed that 2′O-methylation is a Se-dependent process, and methylated tRNA^[Ser]Sec^ is superior to less modified tRNA^[Ser]Sec^ in supporting Sec incorporation into GPX1 and other stress-related selenoproteins. Because selenoprotein expression is generally more stable in females than in males, we speculated that FTSJ1, which is associated with X-linked mental disability in humans [52], might represent the elusive 2′-O-methyltransferase. We recently inactivated the *Ftsj1* gene in mice and demonstrated by mass-spectrometry that 2′O-methylation of U_34_ in tRNA^[Ser]Sec^ is entirely undetectable in tRNA^[Ser]Sec^ isolated from *Ftsj1* mutant mice [53]. This work has included ribosomal profiling of *Ftsj1*-deficient brain, but expression of selenoproteins was not specifically investigated. Here, we subjected the dataset from the earlier study to our analysis pipeline regarding selenoprotein expression (Figure 7). Plotting the density of RPFs around the UGA/Sec codon of all selenoproteins showed absolutely no difference between controls and *Ftsj1*-KO mice (Figure 7A). Calculation of ΔURE likewise showed no differences, in particular, for *Gpx1*, the selenoprotein best known for its response to Um_34_ modification in tRNA^[Ser]Sec^ (Figure 7B). The ribosomal coverage and cumulative sum plots of *Selenop* did not show any impact of the *Ftsj1* inactivation despite 10 UGA/Sec codons in the open reading frame (Figure 7C). Finally, ribosomal coverage of *Gpx1* was not reduced either (Figure 7D).

## 3. Discussion

Expression of selenoproteins is governed by the availability of Se. Dietary Se restriction, interference with Se transport within the body or pathogenic variations in genes encoding certain biosynthesis factors have a major impact on selenoprotein biosynthesis [4]. The above effects all converge on the availability of amino-acylated tRNA^[Ser]Sec^ (Sec-tRNA^[Ser]Sec^). This notion is supported by a hypomorphic mouse model with a promoter mutation in the gene encoding tRNA^[Ser]Sec^ [23].

A large body of evidence suggests that hierarchical expression of selenoproteins is modulated, perhaps governed, by modification of tRNA^[Ser]Sec^. However, most of the studies delineating the function of tRNA^[Ser]Sec^ in selenoprotein expression were based on (over-)expression of mutant tRNA^[Ser]Sec^ in the presence or not of endogenous, functional tRNA^[Ser]Sec^. In particular, the mouse model expressing A37G mutant tRNA^[Ser]Sec^ has been the subject of many studies [30,33]. However, multiple copies of the mutant transgene have integrated into the mouse genome, and a direct effect of the base exchange on tRNA structure may also affect tRNA charging, binding to the elongation factor, or decoding in the ribosome. Hence, we wanted to address the question of tRNA^[Ser]Sec^ modification from the side of the modifying enzyme and studied cell and animal models deficient in the tRNA-isopentenyltransferase TRIT1.

Besides tRNA^[Ser]Sec^, this enzyme modifies several substrates, among them, cytosolic tRNA^Ser^_(UCN)_ and several mitochondrial tRNAs [21,36,42,54]. In fact, patients carrying pathogenic *TRIT1* variants show a mitochondrial phenotype [43,55]. Yet, although the p.R323Q variant greatly diminished TRIT1 activity towards tRNA^[Ser]Sec^ in vitro, we found no evidence that selenoprotein expression was generally reduced in patient fibroblasts. In fact, a deficiency of selenoproteins is usually reflected by an induction of NRF2-target genes [56,57]. In these cells, however, many genes known to be induced by NRF2 in selenoprotein deficiency are not up- but down-regulated.

The most direct way to assess the effect of tRNA isopentenylation in translation of selenoproteins is gene targeting of the responsible enzyme, TRIT1. We have generated conditional *Trit1*-knockout mice and analyzed selenoprotein expression in mouse liver and cultured hepatocytes. In liver, Western blotting showed only SELENOP and SEPHS2 levels moderately reduced, while GPX4 was even increased. We thus used ribosomal profiling to assess translation through UGA/Sec in mouse liver. When we summed up all ribosome protected fragments of selenoproteins with the UGA/Sec codon in the A-site, we found a small decrease in *Trit1*-KO liver. Individual analyses of all selenoproteins expressed in mouse liver supported reduced translation of *Sephs2*, *Selenop, and Txnrd1* after the UGA/Sec codons. GPX1 protein level and *Gpx1* translation were not altered in the *Trit1* mutant. The decrease in GPX1 and the preservation of TXNRD1, however, were among the key observations in the A37G mutant tRNA mouse model. Thus, we conclude that the effect of the A37G mutation does not result from the lack of isopentenylation of base 37, but from the base exchange.

It has been proposed that the A37G mutation in tRNA^[Ser]Sec^ leads to hypomodification of U_34_. Base 34 carries two modifications: mcm^5^U and 2′-O methylation (mcm^5^Um_34_). Since the U34A(I) mutated tRNA^[Ser]Sec^ also shows massively reduced selenoprotein expression, it was concluded that lack of ribose 2′-O methylation was the reason for reduced UGA/Sec translation in both mouse models [31]. In fact, exposure of endothelial cells to an inhibitor of S-adenosylhomocysteine (S-Ado-Hcy) hydrolase reduced both GPX1 and TXNRD1 expression [58]. The authors showed that increased levels of S-Ado-Hcy increased the level of mcm^5^U tRNA^[Ser]Sec^ at the expense of the mcm^5^Um isoform, possibly through inhibition of the elusive 2′O-methyltransferase [58]. Gene targeting in mice showed that FTSJ1 is the 2′O-methylase of U_34_ in tRNA^[Ser]Sec^ [53]. However, our data do not support a role of FTSJ1 in UGA/Sec re-coding during selenoprotein translation in mice fed adequate Se levels in their diet. It is still possible that analysis of mice fed a Se-deficient diet may reveal an effect of Um_34_ in tRNA^[Ser]Sec^ on selenoprotein translation. Apart from this possibility, is there any other way to reconcile these seemingly conflicting observations?

It is interesting that UGA/Sec re-coding in *Gpx1* is not (always) sensitive to full mcm^5^U modification [59]. So, we wonder whether one could look at the available data in another way: formation of mcm^5^Um_34_ in tRNAs is not unique for tRNA^[Ser]Sec^, but may represent a general mechanism to cope with oxidative stress [60]. Impaired expression of selenoproteins, in turn, leads to oxidative stress [61]. Thus, mcm^5^Um methylation and GPX1 activity may correlate, but not necessarily through tRNA modification. This idea would explain why co-administration of the antioxidant N-acetylcysteine with the S-Ado-Hcy hydrolase inhibitor rescued GPX1 expression [58]. An antioxidant should not be able to replace a specific methylase activity.

**Figure 8:**
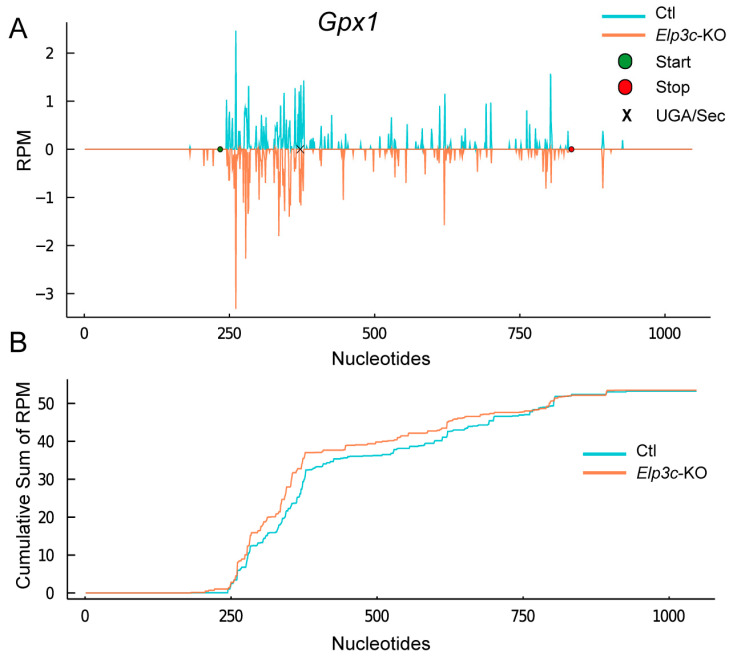
Ribosomal profiling of *Gpx1* in *Elp3* knockout (KO) developing brain. (**A**) Ribosomal coverage plot. The position of the UGA/Sec codon is indicated by a black “x” mark. Note the decreased ribosomal coverage in *Elp3* KO 3′ from the UGA/Sec codon. (**B**) Cumulative sum plot. The net translation of *Gpx1* in *Elp3* KO is apparently adjusted by increased translation/initiation 5′ of the UGA/Sec. Data from [62] were re-analyzed with the methods presented here.

The modification of U_34_ is, in fact, important, as shown by two groups that independently targeted the *Alkbh8* gene in mice [38,39]. ALKBH8 is the methylase forming the methyl-ester in mcm^5^U. Lack of this modification clearly impairs translation of GPX1 in liver and fibroblasts [38,39], while in lungs, TXNRD1 is more affected [59]. We hypothesized that inactivation of the elongator complex, which initiates the mcm^5^U modification, should impair selenoprotein translation. A paper targeting *Elp3* in mouse developing cortex has provided ribosomal profiling data [62]. We analyzed this dataset using the methodology presented here and found that *Gpx1* indeed shows decreased UGA/Sec read-through (Figure 8). Due to the low sequencing depth of this experiment, it is difficult to make statements on less abundantly expressed selenoproteins, and a future experiment should analyze a hepatocyte-specific *Elp3* knockout model.

It is an intriguing observation that interference with different positions in tRNA^[Ser]Sec^ and different modifications leads to very specific effects on the expression of only a subset of selenoproteins: lack of i^6^A_37_ affects TXNRD1 and GPX4, but not GPX1. Inactivation of *Alkbh8* affects GPX1 in liver and fibroblasts, but TXNRD1 in lung. Mutation of A_37_ to G reduces GPX1 expression but not TXNRD1. Research into this mechanism will profit from using the same type of cell or organ and the same methodology. Finally, Se availability may mitigate or potentiate effects of tRNA modification [33]. What remains beyond is the question of how tRNA modification can differentially affect the UGA/Sec re-coding event in different selenoproteins. Here, we are lacking data on the mammalian ribosome in complex with SECISBP2, mRNA, and tRNA^[Ser]Sec^. It is conceivable that codon context, i.e., bases 5′ and 3′ from the UGA will modulate how the codon and the anticodon accommodate in the ribosomal decoding center. Transfer RNA^[Ser]Sec^ modifications may enhance decoding or not, e.g., in the bacterial cryo-EM structure, a hydrogen bond is observed between 2′O-U_34_ and 5′O-C_35_ [35]. Hence, a 2′O methylated U_34_ may be able to modulate codon:anticodon interactions. In the bacterial situation, the two bases following the UGA codon engage in stacking interactions with bases from the 16S ribosomal RNA in which the hypermodified U_34_ is involved. Thus, a sequence-specific communication between codon context and tRNA modification is conceivable and awaits experimental verification.

## 4. Materials and Methods

### 4.1. Mouse Model

The generation and further characterization of the conditional *Trit1* mouse model will be described elsewhere in the context of the impact of TRIT1 on translational fidelity (Bohleber, Fradejas, Suzuki, Schweizer et al., in preparation). Animal experiments were performed according to approval by the LANUV Recklinghausen (AZ 84-02.04.2014.A436 and 81-02.04.2020.A042).

### 4.2. Human Fibroblast Culture

Cells were cultured following the same procedures described [43].

### 4.3. Hepatocyte Culture

Procedure was described in [51]. After perfusion of mice, livers were mechanically disaggregated into DMEM high Glucose, 10% FBS, 1% Glutamine and 1% Penicillin/Streptomycin. Cell suspension was passed through a cell strainer before going through serial centrifugation/resuspension steps. Finally, cells were counted and seeded in collagen-coating plates. Experiments were performed the following day.

### 4.4. Neuron Culture

The procedure was followed as indicated in Beaudoin et al. [63]. The cortices of each pup (P1) were dissected and individually kept in separate Eppendorf tubes with 1 mL dissection medium. After trypsinization for 20 min at 37 °C, trypsin was removed and cortices were washed with plating medium and triturated with a polished Pasteur pipette against a Petri dish. Cells were passed through a cell strainer, counted, and plated with 1 million cells per 100 mm plate coated with poly-L-lysine. The following day medium was replaced with maintenance medium. After two days in culture, Ara-C was added to a final concentration of 5 µM and kept for one day until half of the medium was replaced by fresh maintenance medium. Cultures were used for experiments around 10 days post-plating.

### 4.5. Western Blot

Mouse tissues (liver and cortex) and confluent human fibroblasts were homogenized in RIPA lysis buffer containing protease inhibitors (Roche, Basel, Switzerland). Protein extracts were resolved by 12% SDS-PAGE, transferred to nitrocellulose membranes (GE Healthcare, Chicago, IL, USA) and immunoblotted using the antibodies against listed in Table S1. Detection was performed with horseradish peroxidase-conjugated anti-mouse or anti-rabbit IgG (Jackson ImmunoResearch, West Grove, PA, USA) and an enhanced chemiluminescence detection system (Supersignal West Dura, Thermo Scientific, Waltham, MA, USA) using Fusion Solo detector (Vilber Lourmat Deutschland GmbH, Eberhardzell, Germany).

### 4.6. ^75^Se Labeling

Confluent human fibroblast, neuron, and hepatocyte cultures grown in 100 mm plates were labelled overnight with radioactive sodium selenite (Na_2_[^75^Se]O_3_) (10 µCi/plate). Cells were washed with 1× PBS and lysed in RIPA buffer. Then, 50 µg of lysate were separated by SDS-PAGE (12% gel). Coomassie blue staining was performed before gel drying (Gel dryer Bio-Rad, Bio-Rad, Hercules, CA, USA). Autoradiography was obtained using a BAS-1800 II (Fujifilm, Tokyo, Japan) Phosphoimager.

### 4.7. Transfer RNA Modification Index by RT-PCR

The qualitative determination of the ms^2^i^6^A modification in human mitochondrial tRNAs was adapted from [45]. Total RNA was extracted with Trizol (Invitrogen, Waltham, MA, USA) following the manufacturer’s instructions. DNase treatment and cDNA synthesis were performed according to Xie et al. [45], using RQ1 RNase-free DNase (Promega, Madison, WI, USA) and the Transcriptor First Strand cDNA Synthesis Kit (Roche, Basel, Switzerland). Primers used for cDNA synthesis and qPCR were previously published in [64]. qPCR was performed using Absolute qPCR SYBER Green according to the manufacturer’s instructions in a Mastercycler epgradient S realplex (Eppendorf, Hamburg, Germany). Specific primer annealing temperatures were determined by gradient PCR (see Table S2). Amplified products were verified by melting curve analysis and gel electrophoresis. Modification indexes were calculated as in [45].

### 4.8. TRIT1 In Vitro Assay

Human *TRIT1* gene was cloned, the p.R323Q variant introduced, and recombinant protein (wild type or variant TRIT1) purified using the same methods as for the mouse TRIT1 in [42]. Primers used for cloning and site direct mutagenesis are shown in Table S3. Reactions were performed using the same conditions as in [42], but with slight variations. Different anticodon stem loop RNA primers were used as substrate (Table S4). Then, 2.5 U of pyrophosphatase (Genecraft, Cologne, Germany) were added to the mixture. The reaction was stopped after 10 min by adding 100 µL of ice-cold 10% TCA. Precipitation was done via a modified TCA precipitation protocol [65]. The whole volume of reaction tube was transferred to a Whatman filter paper and air dried for 15 min. Afterwards, it was washed in TCA (10%), in EtOH (95%), and in diethyl ether. The filter paper was air dried for 30 min between the washing steps and for 60 min after the last one. Scintillation liquid was added to the filter papers and a measurement was measured in a LS 6500 scintillation counter (Beckman, Pasadena, CA, USA).

### 4.9. tRNA^[Ser]Sec^ Northern Blot

The procedure and probes used were previously described [51].

### 4.10. Quantification of i^6^A by LC-MS

First, 500 ng tRNA were digested into nucleotides using 0.3 U nuclease P1 from *P. citrinum* (Sigma-Aldrich, St. Louis, MI, USA), 0.1 U snake venom phosphodiesterase from *C. adamanteus* (Worthington, Columbus, OH, USA), 200 ng Pentostatin (Sigma-Aldrich, St. Louis, MI, USA), and 500 ng Tetrahydrouridine (Merck-Millipore, Burlington, MA, USA) in 5 mM ammonium acetate (pH 5.3; Sigma-Aldrich, St. Louis, MI, USA) for two hours at 37 °C. The remaining phosphates were removed by 1 U FastAP (Thermo Scientific, Waltham, MA, USA) in 10 mM ammonium acetate (pH 8) for one hour at 37 °C. The nucleosides were then spiked with internal standard (^13^C stable isotope-labeled nucleosides from *E. coli*, SIL-IS) and subjected to analysis. Technical triplicates with 26.6 ng digested RNA and 20 ng internal standard were analyzed via LC–MS (Agilent 1260 series and Agilent 6460 Triple Quadrupole mass spectrometer equipped with an electrospray ion source (ESI)). The solvents consisted of 5 mM ammonium acetate buffer (pH 5.3; solvent A) and LC–MS grade acetonitrile (solvent B; Honeywell, Charlotte, NC, USA). The elution started with 100% solvent A with a flow rate of 0.35 mL/min, followed by a linear gradient to 10% solvent B at 20 min, 25% solvent B at 30 min and 80% solvent B after 40 min. Initial conditions were regenerated with 100% solvent A for 14 min. The column used was a Synergi Fusion (4 µM particle size, 80 Å pore size, 250 × 2.0 mm; Phenomenex, Torrance CA, USA). The UV signal at 254 nm was recorded via a diode array detector (DAD) to monitor the main nucleosides. ESI parameters were as follows: gas temperature 350 °C, gas flow 8 L/min, nebulizer pressure 50 psi, sheath gas temperature 300 °C, sheath gas flow 12 L/min, and capillary voltage 3500 V. The MS was operated in the positive ion mode using Agilent MassHunter software in the dynamic MRM (multiple reaction monitoring) mode. For relative quantification, the signals of i^6^A were normalized to the ^13^C-labeled signal and then normalized to the UV signal of guanosine.

### 4.11. Isolation and LC/MS Analysis of tRNA^[Ser]Sec^

Mouse liver total RNA was separated by anion exchange chromatography with DEAE Sepharose Fast Flow (GE Healthcare, Chicago, IL, USA) to obtain crude tRNAs with removal of polysaccharides and rRNA [66]. Cytoplasmic tRNA^[Ser]Sec^ was isolated from the crude tRNAs by reciprocal circulating chromatography, as described in [48]. The 5′-EC amino-modified DNA probe (Sigma-Aldrich, St. Louis, MI, USA), TGGGCCCGAAAGGTGGAATTGAACCACTCTGTCGCTAGAC was covalently immobilized on NHS-activated Sepharose 4 Fast Flow (GE Healthcare, Chicago, IL, USA). About 6 μg of tRNA^[Ser]Sec^ were obtained from 1.4 mg crude tRNAs. Mouse tRNA^[Ser]Sec^ was digested by RNase T1 (Thermo Fisher Scientific, Waltham, MA, USA), and subjected to capillary liquid chromatography (LC) coupled to nano electrospray (ESI)/mass spectrometry (MS) on a linear ion trap-Orbitrap hybrid mass spectrometer (LTQ Orbitrap XL; Thermo Fisher Scientific, Waltham, MA, USA), as described in [49,50]. The RNA fragments were scanned in a negative polarity mode over a range of *m*/*z* 600–2000.

### 4.12. 3′-. RNA Sequencing

RNA was extracted from human fibroblasts with TRIzol Reagent (Invitrogen, Waltham, MA, USA) according to the manufacturer protocol. Approximately, 500 ng of RNA were used for library preparation with QuantSeq 3′-mRNA Library Prep (Lexogen, Vienna, Austria). Sequencing was performed by the Illumina HiSeq 2500 instrument on 50-cycle single-end mode.

### 4.13. RiboSeq

Treatment of the samples was performed as described previously [47,51], with some changes for generating the RPF. Cycloheximide was omitted from the lysis buffer. All steps were carried out on ice. Then, 50 mg of frozen mouse liver was crushed in 1000 μL ice-cold lysis buffer using a pellet pestle. Lysate was pipetted 3 times up and down with a 1000 μL pipette before it was passed through a 26-gauge needle for 10 times. After 10 min incubation on ice, the lysate was centrifuged at 20.000× *g* for 10 min at 4 °C. The supernatant was transferred to a new 1.5 mL reaction tube. Then, 200 μL of the lysate were incubated for 60 min with 1000 U of RNase I at 25 °C and at 1300 rpm in a thermoblock. Pre-processing and alignments of the reads were performed as described before [47]. For analysis, 28 nt and 29 nt read sizes and an offset of 12 nt to the P-site were used. Three individual mouse livers were used per genotype.

## Figures and Tables

**Table 1:** Determination of kinetic parameters of recombinant human TRIT1 with anticodon-containing fragment substrates.

	tRNA	K_M_ [µM]	V_max_ [pmol/min * mg Protein]	Sequence
cytosolic	Ser AGA	0.7980 ± 0.1091	989.0 ± 45.27	GA-UGG-ACU-AGA-AAU-CCA-UU
	Ser CGA	0.4384 ± 0.0849	465.5 ± 27.17	GU-UGG-ACU-CGA-AAU-CCA-AU
	Ser UGA	0.8690 ± 0.1210	1016 ± 54.85	GA-UGG-ACU-UGA-AAU-CCA-UU
	Sec UCA	0.3848 ± 0.0934	321.5 ± 25.72	UG-CAG-GCU-UCA-AAC-CUG-UA
mitochondrial	Cys GCA	5.293 ± 4.294	73.94 ± 28.37	AU-UGA-AUU-GCA-AAU-UCG-AA
	Ser UGA	1.673 ± 0.2683	523.6 ± 32.23	GG-UUG-GCU-UGA-AAC-CAG-CU
	Trp UCA	3.710 ± 0.5627	442.2 ± 31.37	AA-GAG-CCU-UCA-AAG-CCC-UC
	Tyr GUA	0.7735 ± 0.1127	549.7 ± 26.37	AU-UGG-ACU-GUA-AAU-CUA-AA

## Data Availability

The ribosomal profiling data on *Trit1*-KO liver was submitted to GEO (GSE183923).

## Supplementary Materials

All data are available online at https://www.mdpi.com/article/10.3390/ijms222111454/s1.
