# Supplementary material for: The Effect of tRNA[Ser]Sec Isopentenylation on Selenoprotein Expression
Source: Int J Mol Sci. 2021 Oct 23;22(21):11454. doi: 10.3390/ijms222111454 (PMC8583801; doi:10.3390/ijms222111454)
Supplement: Supplementary file 1 [file ijms-22-11454-s001.zip › ijms-1414237-supplementary.pdf]

Table S1: Antibodies used in this study

Antibody	Host species	Dilution	Manufacturer	Catalogue number
TRIT1	Rabbit	1:1000	Sigma	HPA024174
TRIT1	Rabbit	1:500	Novus Biologicals	NBP2-20727
β-ACTIN	Mouse	1:25000	Sigma	A3854
α-TUBULIN	Rabbit	1:2500	Rockland	600-401-880
GPX1	Rabbit	1:1000	Abcam	ab22604
GPX4	Rabbit	1:1000	Abcam	ab125066
SELENOT	Rabbit	1:250	Sigma	HPA039780
TXNRD1	Mouse	1:1000	Abcam	ab16847
SELENOK	Mouse	1:500	Sigma	HPA008196
SELENOS	Rabbit	1:1000	Sigma	HPA010025
SELENOF	Rabbit	1:5000	Abcam	ab124840
SELENOW	Rabbit	1:2000	Rockland	600401A29
SELENOP	Rabbit	1:400	Immunoglobe	0122-03
SEPHS2	Rabbit	1:2000	Rockland	200-401-999
HRP goat anti mouse	Goat	1:1000	Jackson Immunotech (Dianova)	115-035-003
HRP goat anti rabbit	Goat	1:1000	Jackson Immunotech (Dianova)	111-035-003

Table S2: Specific annealing temperatures used to determine modification index by qPCR

	**tRNA**	**Annealing temperature**
Human	mt-tRNA^{Phe}	58.3°C
	mt-tRNA$^{Ser(UCN)}$	59.2°C
	mt-tRNA^{Tyr}	60.2°C
	mt-tRNA^{Trp}	62°C
	mt-tRNA^{Cys}	61°C

Table S3. Primers used for *TRIT1* cloning, site-direct mutagenesis to introduce R323Q patient mutation

**Purpose**	**Primer**	**Sequence (5'-3')**
***TRIT1* cloning**	hTRIT1_Fw_NcoI	ACTGCCATGGCGTCCGTGGCGGCTGCACGAGCAG
	hTrit1_Rv_His	CAGTCCTAGGTTAATTAGTGGTGGTGATGGTGATGATGGTGGTGATGAGCGGAAACGCTGCATTTCAGCTCTTGATC
**R323Q point mutation**	hTRIT11g982a_Fw	GTAACTAAGAGATATGCCCAGAAACAAAACCGATGGGTT
	hTRIT1g982a_Rv	AACCCATCGGTTTTGTTTCTGGGCATATCTCTTAGTTAC

Table S4: Anticodon stem loop (ASL) RNA primers used for TRIT1 assay. Mutated A>G Sec ASL at position 37 (in bold) was used as negative control.

	**RNA Primer**	**Sequence (5'-3')**
**Cytosolic ASL**	Ser (UGA)	GA-UGG-ACU-UGA-AAU-CCA-UU
	Ser (AGA)	GA-UGG-ACU-AGA-AAU-CCA-UU
	Ser (CGA)	GU-UGG-ACU-CGA-AAU-CCA-AU
	Sec (UCA)	UG-CAG-GCU-UCA-AAC-CUG-UA
**Mitochondrial ASL**	Cys (GCA)	AU-UGA-AUU-GCA-AAU-UCG-AA
	Ser (UGA)	GG-UUG-GCU-UGA-AAC-CAG-CU
	Trp (UCA)	AA-GAG-CCU-UCA-AAG-CCC-UC
	Tyr (GUA)	AU-UGG-ACU-GUA-AAU-CUA-AA
**Negative control**	Sec (UCA) A37G	UG-CAG-GCU-UCA-**G**AC-CUG-UA
